# Hydroxysafflor yellow A acutely attenuates blood-brain barrier permeability, oxidative stress, inflammation and apoptosis in traumatic brain injury in rats^1^

**DOI:** 10.1590/ACB351202

**Published:** 2021-01-20

**Authors:** Jianjun Xu, Tian Zhan, Wan Zheng, Yun-Ke Huang, Ken Chen, Xian-Hua Zhang, Ping Ren, Xi Huang

**Affiliations:** I Master, Nanjing University of Chinese Medicine, China.; II Master, Department of TCM-related comorbid depression, Nanjing University of Chinese Medicine, China.; III PhD, Nanjing University of Chinese Medicine, China.; IV Master, Women’s Hospital School of Medicine Zhejiang University, China.; V Master, Department of TCM-related comorbid depression, Nanjing University of Chinese Medicine, China.; VI PhD, Xiamen Mental Health Center, Xiamen Xianyue Hospital, China.; VII PhD, Department of Geriatrics, Affiliated hospital Nanjing University of Chinses Medicine, China.; VIII PhD, Department of TCM-related comorbid depression, Nanjing University of Chinese Medicine, China.

**Keywords:** Brain Injuries, Traumatic, Blood-Brain Barrier, Oxidative Stress, Apoptosis, Rats

## Abstract

**Purpose:**

To investigate the therapeutic benefits of Hydroxysafflor yellow A (HSYA) on blood-brain barrier (BBB) vulnerability after traumatic brain injury (TBI) and identify its potential action of mechanisms on TBIinduced injuries.

**Methods:**

The rat TBI model was performed by using a controlled cortical impact device. The BBB permeability induced by TBI was measured through Evans Blue dye superflux and western blotting or polymerase chain reaction (PCR) for tight junctional proteins (TJPs). The post-TBI changes in oxidative stress markers, inflammatory response and neuron apoptosis in brain tissue were also tested.

**Results:**

Herein, the results showed that HSYA acutely attenuated BBB permeability via increasing the production of the TJPs, including occludin, claudin-1 and zonula occludens protein 24 h after TBI. Additionally, HSYA could suppress the secretion of proinflammatory factors, such as interleukin-1β, interleukin-6, and tumor necrosis factor-α (IL-1β, IL-6, and TNF-α), and also concurrently down-regulate the expression of inflammation-related Toll-like receptor 4/nuclear factor kappa-B (TLR4/NF-kB) protein. These HSYA challenged changes were accompanied by the decreased TBI induced oxidative stress markers and inhibited the expression of apoptosis proteins Bax, caspase-3 and caspase-9.

**Conclusions:**

Taken together, all findings suggested that HSYA (30 mg/kg) are against TBI through improving the integrity in BBB, which are associated with the antioxidant, anti-inflammation and antiapoptosis via the probable mechanism of down-regulation of the TLR4/NF-kB pathway, and its in-detail protective mechanisms are under study.

## Introduction

Traumatic brain injury (TBI) is an important contributor to worldwide mortality and morbidity, especially in young people, and is closely associated with decreased life expectancy^1^. It is also a crucial medical, public health and socioeconomic problem around the world^1^. Pathophysiology of TBI involves two general stages: primary and secondary brain injury. After TBI, oxidative stress is generated and then antioxidant enzymes decrease, resulting in neural dysfunction and cell death^2^. Moreover, recent observations showed that inflammation is closely related to the etiology and pathogenic mechanism of TBI, which is a crucial contributor to second brain damage. Toll-like receptor 4 (TLR4) could accelerate the secretion of various proinflammatory factors (IL-1, IL-6 and TNF-α) after combined with high-mobility group box-1 (HMGB1) and could also rise enhanced secretion and release of proinflammatory cytokines via up-regulation of nuclear factor kappa-B (NF-κB)^3^.

Tight junctions proteins (TJPs) are critical to the integrity of the blood-brain barrier (BBB), including the occludin, claudin and zonula occludens protein (ZO-1)^4^–^6^. Overexpression of the occluding and claudin in TJ strands of endothelial cells can be contributed to increase BBB integrity^7^^,^ 8. Moreover, inflammation and oxidative stress both involved in changing BBB integrity against TBI^4^, especially several activated proinflammatory cytokines, such as IL-1, which could reduce the level of TJPs or accelerate junctional disintegration of endothelia claudin and occludin under various neuroinflammatory disease, thereby increasing the BBB permeability^8^^,^ 9. Therefore, an effective therapeutic strategy that can alleviate the brain damage against TBI through inhibiting oxidative stress, inflammatory, increase the BBB integrity and recovery functions is urgently needed.

*Carthamus tinctorius* L. from Danshen-Chuanxiong-Honghua is a traditional Chinese medicine, which was widely used for the treatment of cerebrovascular and cardiovascular diseases for thousands of years^10^. Hydroxysafflor yellow A (HSYA), a primary active and representative component of *Carthamus tinctorius L,* has been demonstrated to possess broad pharmacological functions, especially antioxidant stress and anti-inflammatory activity^10^–^13^. Previous studies suggested that HSYA features greatest neuroprotection in animal models of cerebral I/R injury^14^, spinal cord compression injury^15^ and Parkinson’s disease^16^. However, the detailed effects and the underlying mechanisms of HSYA on TBI remain unclear. On this basis, the results tested a hypothesis that HSYA can benefit BBB vulnerability of TBI with a particular focus on its antioxidation, anti-inflammatory and anti-apoptosis effects. The results would provide evidence that HSYA may help therapeutic strategy against TBI.

## Methods

### Reagents

Hydroxysafflor yellow A, with purity > 98%, were obtained from Shanghai Yuanye Biotechnology Co., Ltd. Evans blue was purchased from Beijing Solarbio Science & Technology Co., Ltd. Rabbit monoclonal ZO-1 antibody (1:500), rabbit monoclonal occludin antibody (1:500), rabbit monoclonal Bax antibody (1:2000), rabbit monoclonal IL-6 antibody (1:1000), rabbit monoclonal TLR4 antibody (1:500), mouse monoclonal TNF-a antibody (1:1000), mouse monoclonal Caspase-3 antibody (1:1000), mousemonoclonal Caspase-9 antibody (1:500) and mouse β-actin antibody (1:5000) were purchased from Proteintech Group. Rabbit monoclonal IL-1β antibody (1:1000), rabbit monoclonal NF-κB antibody (1:50000) and rabbit monoclonal Claudin-1 antibody (1:2000) were purchased from Abcam. All secondary antibodies were gained from Proteintech Group, Inc.

### Animals and experiment design

Male Sprague-Dawley (SD) rats (200 - 250 g) were purchased from Laboratory Animal Co. Ltd of JieSiJie (Shanghai, China) and lived in the laboratory (23 ± 2 °C; 12/12 h dark/light cycle; 50 ± 10% humidity) with free access to standard food and water for one week prior tothe study. All animals were kept in strict accordance with the National Laboratory Animal Management Regulations and guidelines of the Animal Feeding and Ethics Committees of the Experimental Animal Center of Nanjing University of Chinese Medicine (Laboratory Animal Ethics No.: 201912A017).

The rats were randomly divided into the three groups in this experiment, as follows: i) sham group: rats were orally administered with saline (0.9% NaCl) and experienced the same operative procedures, but head impactor was not released; ii) vehicle group: rats with controlled cortical impact (CCI) were also orally administered with saline (0.9% NaCl); and iii) HSYA treatment group: rats were single orally administered with HSYA following trauma^13^, which was dissolved in saline (0.9% NaCl) with ultrasound for 5 min. The dose of HSYA was 30 mg/kg, according to the previous study^13^, and its content from parent formula Danshen-Chuanxiong-Honghua(3.101 ± 0.026 mg/g, not reported). The administration volume in all experiments was 1.0 mL/100 g.

### Traumatic brain injury model

The CCI model with TBI was operated using an electronic controlled pneumatic impact device (TBI 0310; Precision Systems and Instrumentation LLC, Fairfax Station, VA, USA), which was composed of a hard stop Bimba cylinder (Bimba Manufacturing, Monee, IL, USA) and an impactor cusp (outside diameter, 5 mm)^17^. Surgical anesthesia was developed under 4% isoflurane with 70% N_2_O and 30% O_2_. All rats were immobilized in a stereotaxic frame and their scalp were retracted to expose the skull under maintenance of 2% isoflurane inhalation. After a 5-mm diameter hole was drilled on the right cerebral hemisphere, animals were subjected to CCI at 5 m/s impact velocity, 5 mm depth and 2000 msec dwell time. Thereafter, a warming pad was used to maintain constant normothermia.

### Measurement of BBB permeability

The permeability of BBB was measured by the superflux of Evans blue (EB) dye, as previously described^18^, 12 h after CCI damage. Evans blue dye (2% in saline) was injected slowly into rats via caudal vein (3 mL/kg) and let to circulate for 1 h. Subsequently, animals were anesthetized with 1% pentobarbital sodium and transcardially perfused with saline. Then, the injured hemisphere was collected, dried, weighed and hatched in formamide (4.0 mL) at 60 °C for 24 h. After incubation, the supernatants were obtained by centrifugation for 15 min at 3000 rpm (r_max_ = 82.5 mm). Finally, the optical density (OD) values were detected at 620 nm to calculate the quantity of extravasated EB dye in each sample, according to the standard curve.

### Measurement of oxidative stress levels

Rats were decapitated 24 h post-CCI damage under anesthesia. The hippocampus and cortex^13^ were isolated from the injury ipsilateral brain, frozen and stored at -80 °C.The frozen cortex was weighed and homogenized in ice-cold saline (1:9, w/v). The homogenates were centrifuged at 3000 rpm (r_max_=82.5 mm) at 4 °C for 10 min and then the supernatants were used to evaluate the oxidative indices with the reagent kits (Nanjing Jiancheng Bioengineering Institute).

### Quantitative real-time PCR analysis

Total RNA extracted from the ipsilateral hippocampus of post-TBI rats using TRIzol reagent (Invitrogen, Grand Island, USA). The single-stranded cDNA was synthesized through reverse transcribe of total RNA, according to the reverse transcriptase kit (Beijing, China). All gene-specific mRNA levels were measured by quantitative real-time PCR (RT-qPCR). The reaction was run under the following conditions: initial denaturation at 95 °C for10 min, followed by 40 cycles of 95 °C for 15 s and 60 °C for 50 s. The primer sequences were presented in Table 1. Relative mRNA expression levels were evaluated by the 2^-ΔΔCt^ method. All results were normalized to β-actin gene.

**Table 1:** Primer sequences for RT-qPCR.

Gene	Forward sequence	Reverse sequence
ZO-1	5’- ACAGCCAGCTCTTGGTCAT -3’	5’- GTATGGTGGCTGCTCAAGGT -3’
Occludin	5’- CTACTCCTCCAACGGCAAAG -3’	5’- AGTCATCCACGGACAAGGTC -3’
claudin-1	5’- CCCCAATGGAAGATTTACTCCT -3’	5’- GTATCTGCCCGGTGCTTT -3’
β-actin	5’- ACATCCGTAAAGACCTCTATGCC -3’	5’- CTCCTGCTTGCTGATCCAC -3’

### Western blot analysis

The western blot analysis was conducted according to a previously reported method^19^. The concentration of extracted proteins was measured by BCA Protein Quantification Kit (wellbio). After denaturation in boiling water, protein samples (30 μg) were loaded onto polyacrylamide gel and separated. Following electrophoresis, the proteins were transferred onto polyvinylidene difluoride (PVDF) membrane, and then the membranes were blocked with Tris-buffered saline Tween-20(TBST) containing 5% skimmed milk powder at room temperature for 1.5 h. Subsequently, the membranes were incubated with each primary antibody against IL-1β (1:1000), IL-6 (1:1000), TNF-α (1:1000), TLR4 (1:500), NF-κB (1:50000), Bax (1:2000), casapase-3 (1:1000), casapase-9 (1:500), ZO-1 (1:500), occludin (1:500) and Claudin-1 (1:2000) overnight at 4 °C. After three washings with TBST for 15 min each, the membranes were incubated with the appropriate horseradish peroxidase (HRP) conjugated goat anti-rabbit IgG (1:6000, Proteintech Group) or HRP conjugated goat anti-mouse IgG (1:5000, Proteintech Group) for 1 h at room temperature. Immunoreactivity was reacted with a PierceTM ECL substrate (thermo) for 3 min and then exposed to X-ray films. The results were standardized to the intensity levels of β-actin and analyzed using the Quantity-One software (Bio-Rad, Hercules, CA, United States).

### Statistical analysis

All data were analyzed using GraphPad Prism 5 software (San Diego, CA, USA). The significant difference between three groups was determined by one-way ANOVA with Tukey’ s multiple comparisons. The values of p < 0.05 were considered statistically significant. All data of this study are presented as the mean ± SD.

## Results

### Effect of HSYA on BBB permeability in TBI rats

**Figure 1:**
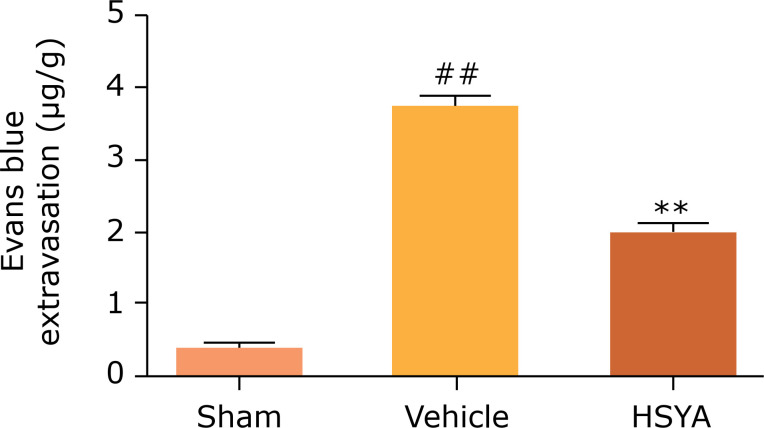
Effects of HYSA on BBB permeability in rats after TBI. Blood-brain Barrier permeability was determined and quantified 12 h post-TBI by Evans blue extravasation, compared with the vehicle group. N = 6 per group; data are presented as mean ± SD;

To examine the protective effect of HSYA on BBB disruption in CCI rats, an extravasation test of Evans blue was performed 12 h post-injury. Figure 1 indicates that CCI injury significantly increased the EB content in the lesions hemisphere and worsen the BBB permeability compared with the sham group (p < 0.01). On the contrary, HSYA treatment (30 mg/kg) enormously decreased the EB level compared with the vehicle group (p < 0.01). The results show that HSYA treatment can improve the function outcome of BBB after CCI injury.

### Effects of HSYA on the TJPs in TBI rats

**Figure 2:**
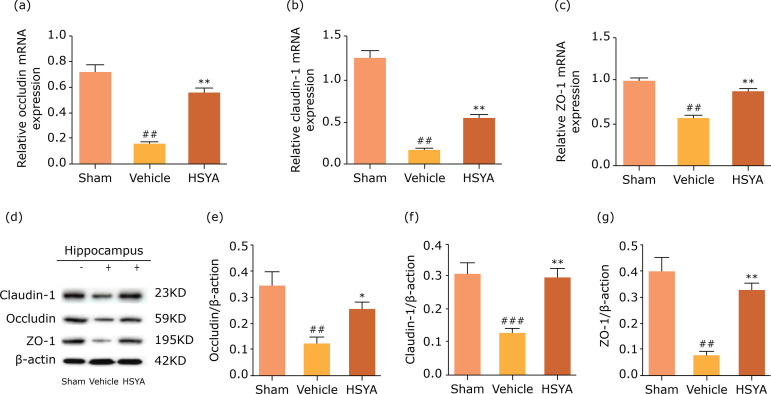
Hydroxysafflor yellow A improved BBB integrity and increased the transmission of junctional proteins in lesion hippocampus of TBI rats. **(a-c)** The quantity of occludin, claudin-1 and ZO-1 by PCR; **(d)** Representative bands of western blot for occludin, claudin-1 and ZO-1; **(e-g)** The quantity of occludin, claudin-1 and ZO-1 by western bolt. Quantified results were revised by β-actin expression. N = 4 per group; data are presented as mean ± SD;

In the present study, The effect of HSYA on the function of TJPs involved in BBB permeability following TBI injury 24 hwas investigated by western blot and real-time PCR. As shown in Fig. 2, the protein and mRNA levels of occludin, claudin-1 and ZO-1 in lesion hippocampus of TBI rats was obviously reduced when compared to the sham group. However, these levels were both significantly enhanced by HSYA treatment.

### Effects of HSYA on oxidative stress in TBI rats

**Figure 3:**
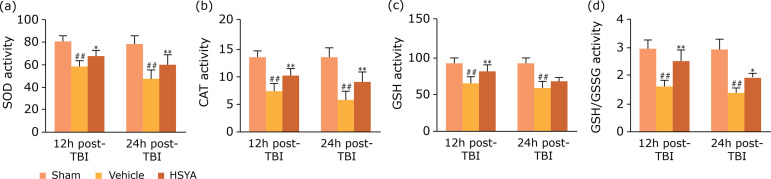
Effects of HSYA on oxidative stress markers in injured cortex at 12 and 24 h after TBI. The levels of (a) SOD, (b) CAT, (c) GSH, (d) ratio GSH/GSSG in injured cortex of TBI rats. N = 6 per group; data are presented as mean ± SD;

The effect of HSYA orally administrated on oxidative stress is represented in Fig. 3. The levels of enzymatic activity of superoxide dismutase, catalase, glutathione, and ratio glutathione/glutathione oxidized (SOD, CAT,GSH and ratio GSH/GSSG) were determined 12 and 24 h after TBI injury. In the vehicle group, TBI obviously reduced the levels of SOD, CAT, GSH and ratio GSH/GSSG in injured cortex when compared with the sham group and was well reversed by HSYA treatment.

### Effects of HSYA on inflammation and apoptosis reaction in TBI rats

**Figure 4:**
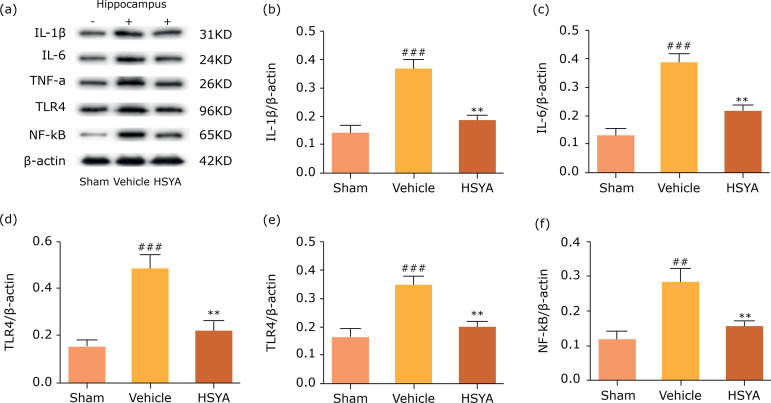
Hydroxysafflor yellow A inhibited protein expression of proinflammatory cytokine and TLR4/NF-kB protein in ipsilateral hippocampus of TBI rats. **(a)** Representative bands of western blot for IL-1β, IL-6, TNF-α, TLR4 and NF-κBprotein levels in sham, vehicle and HSYA group 24 h after TBI; **(b-f)** quantification of IL-1β, IL-6, TNF-α, TLR4 andNF-κB proteins are presented. Quantified results were revised by β-actin expression. N = 4 per group; data are presented as mean ± SD;

**Figure 5:**
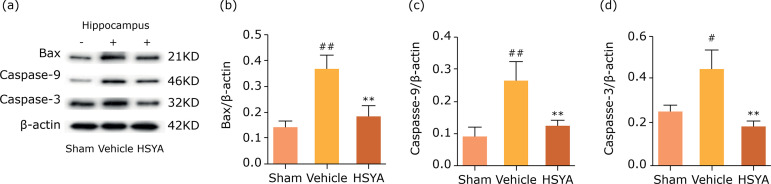
Hydroxysafflor yellow A alleviated neuronal apoptosis in ipsilateral hippocampus of TBI rats. **(a)** Representative bands of western blot for Bax, cleaved caspase-9 and caspase-3 protein levels in sham, vehicle and HSYA group 24 h after TBI; **(b-d)** quantification of bax, cleaved caspase-9 and caspase-3 protein are presented. Quantified results were revised by β-actin expression. N = 4 per group; data are presented as mean ± SD;

The previous study suggests that the inflammation plays an important role in TBI rats and was known closely related to the activity of apoptosis proteins, which contributed to neuronal death and loss of brain tissue^20^. As shown in Fig. 4, the results testified significant increase in protein levels of IL-1β, IL-6, TNF-α, TLR4 and the activation of NF-κB. However, all which were obviously reversed by HSYA treatment in injured hippocampus compared with the vehicle group. Besides, the Bax, caspase-9 and caspase-3 protein levels in lesion hippocampus of rats following TBI was undoubtedly up-regulated; however, these were also counteracted by HSYA treatment in Fig. 5. These results showed that TBI could induce inflammatory response and apoptosis-associated reaction in the injured hippocampus and this outcome could be substantially ameliorated by HSYA treatment.

## Discussion

The findings indicated that HSYA treatment altered BBB permeability with the up-regulation of the TJPs occluding, claudin-1 and ZO-1, preventing aggravation of inflammatory in injured brain. The present study also showed that the beneficial results of HSYA treatment against TBI were concerned with elevated levels of antioxidant defense enzymes (SOD, CAT, GSH, ratio GSH/GSSG), as well as decreased expression of proinflammatory factors (IL-1β, IL-6, TNF-α) and TLR4/NF-κB proteins, resulting in further BBB integrity and potential neuroprotective effects. Besides, HSYA significantly abrogated TBI-induced apoptosis related proteins bax, caspase-3 and caspase-9. These preliminary data concluded that HSYA is applied to treat TBI via elevations in BBB integrity by alleviating oxidative stress, inflammatory response, suppressing caspase-relevant apoptosis.

Oxidative stress, neuroinflammation and apoptosis are regarded as the key pathophysiology of TBI. In secondary damage development, oxidative stress can cause inflammation or be exacerbated by inflammation response^19^. In the present study, HSYA treatment against TBI were concerned with increased levels of GSH and the ratio of GSH/GSSG, as well as activation of antioxidant enzymes (SOD, CAT) (Fig. 3), found in agreement with the mechanism of TBI reported earlier^21^^,^ 22. Apart from oxidative stress, TLR4, one of main receptors related to inflammation and the pathophysiology of secondary injury following TBI, could trigger the activation of pro-inflammatory signaling pathway, such as NF-κB activation, and subsequently the release of proinflammatory cytokines, such as IL-1β and IL-6^3^^,^ 21. Here, the results showed that post-trauma induced TLR4 stimulated NF-κB activity, resulting in the generation of proinflammatory cytokines (IL-1β, IL-6, TNF-α), which promotes the neuroinflammatory response (Fig. 4). All which were successively reversed by HSYA-treatment, suggesting that HSYA can improve the inflammatory response via down-regulation of TLR4/NF-κB pathway.

Additionally, the previous studies showed that pro-inflammatory factors, especially IL-1β, can excite NF-κB nuclear transcription and enhance p53-upregulated modulators of apoptosis, resulting in cell apoptosis and structural brain damage^23^^,^ 24. As an accelerator of apoptosis, Bax is activated by p53 gene, very important in indicator whether the tissue will undergo apoptosis after TBI. Moreover, increasing evidence revealed that an obvious enhancement in both cleaved caspase-9 and caspase-3 activated the mitochondrial apoptosis pathway, ultimately leading to a deterioration in apoptosis in injured brain following TBI^25^. Overall, all findings demonstrated that the therapeutic benefits of HSYA treatment against post-TBI induced neuronal apoptosis is associated with the inhibiting inflammatory response, which down-regulated the expression of TLR4/NF-kB protein and subsequently suppressed inflammation (IL-1β, IL-6 and TNF-α), further resulting in the down-regulation of bax, cleaved caspase-9 and caspase-3 in the ipsilateral hippocampus of rats post-TBI.

Accumulated evidences revealed that post-TBI induced inflammation loses the BBB integrity with remodeling of TJPs, including occluding, claudins and ZO-1^26^. As an integral plasma-membrane protein, occludin is located at the tight junctions, which participate in regulating of tight junction stability and BBB function^27^. Claudins, similar to the tight junction proteins occludin in structure, are the major members of the tight junction proteins, which play an important role in maintaining of the permeability of epithelia. Zonula occludens protein, a tight junction phosphoprotein, localized in intercellular contacts and regulates the BBB integrity, which acts as a molecular scaffold binding various tight junction proteins, including occludin and claudins^28^. All TJPs participated in regulating tight junction stability and BBB function, and played an important role in maintaining the permeability of epithelia^3^. Here, post-trauma tissue resulted in BBB dysfunction can be ameliorated by HSYA by significantly down-regulating the expression of them. Besides, the previous study showed that oxidative stress and inflammatory cytokines from overactivated microglia cells are also involved in BBB dysfunction, modification of TJ integrity. Toll-like receptor 4 could activate microglia to induce release of inflammatory cytokines, such as IL-1β, IL-6 and TNF-α, further increasing BBB damage with concomitant decrease of TJPs after combined with HMGB1^3^. Moreover, along with the disruption of BBB, the migration of neutrophils and mast cells through the endothelial BBB gets easier in post-TBI, further producing a variety of proinflammatory molecule, such as IL-1β, IL-6 and TNF-α, which aggravates the BBB leaking and neuronal apoptosis^29^.

However, the indispensable neuroprotective mechanisms, including infarction volume and/or survival neurons, are unavailable in the present study and are worth further studies to establish the causality of therapeutic benefits and protective mechanism by HSYA. Even so, HSYA helps to find new lead compound against TBI.

## Conclusions

The HSYA treatment might provide an effective prevention and treatment strategy against TBI through improving the BBB integrity with the up-regulation of the TJPs occluding, claudin-1 and ZO-1, and the mechanisms were related to the increase of oxidative stress, the inhibition of the proinflammatory cytokines (IL-1β, IL-6, TNF-α) and neuronal apoptosis, probably via the down-regulation of TLR4/NF-kB pathway.
